# Effects of Airspeed on the Respiratory Rate, Rectal Temperature, and Immunity Parameters of Dairy Calves Housed Individually in an Axial-Fan-Ventilated Barn

**DOI:** 10.3390/ani11020354

**Published:** 2021-01-31

**Authors:** Wanying Zhao, Christopher Choi, Dapeng Li, Geqi Yan, Hao Li, Zhengxiang Shi

**Affiliations:** 1 Department of Agricultural Structure and Bioenvironmental Engineering, College of Water Resources & Civil Engineering, China Agricultural University, Beijing 100083, China; wanyingzhao@cau.edu.cn (W.Z.); lidp@cau.edu.cn (D.L.); yangeqi@cau.edu.cn (G.Y.); leehcn@hotmail.com (H.L.); 2 Key Laboratory of Agricultural Engineering in Structure and Environment, Ministry of Agriculture and Rural Affairs, Beijing 100083, China; 3 Beijing Engineering Research Center on Animal Healthy Environment, Beijing 100083, China; 4 Department of Biological Systems Engineering, University of Wisconsin-Madison, 460 Henry Mall, Madison, WI 53706, USA; cchoi22@wisc.edu

**Keywords:** rectal temperature, respiratory rate, immunity parameters, airspeed, calf

## Abstract

**Simple Summary:**

The practice of raising dairy calves individually has become widespread because it helps dairy farmers guard against the spread of infectious diseases. However, a number of other health issues are associated with the insufficient airflow that develops in some of the interior spaces of a barn divided into individual stalls, especially when the stalls are separated by solid partitions. Typically, a barn’s ventilation fans are arranged in a way that will propel air in a direction perpendicular to the rows of individual compartments, but such an arrangement tends to impede airflow, because the solid partitions that separate the first few stalls deflect the airflow away from stalls further downstream. As a result, only those calves in stalls nearest the fans benefit fully from the airflow’s cooling effect. To determine just how much this disparity might adversely affect those calves in stalls farther from the source of the airflow, we compared the immunity levels of a set of calves housed long term at different distances from the airflow source and under different airflow speeds. We also compared the calves’ respiratory rates and rectal temperatures.

**Abstract:**

At many modern dairy farms, calves raised in barns are kept in individual stalls separated by solid partitions, which act as barriers. Ventilation fans blowing air perpendicular to these stalls only provide the optimal airflow to the first few calves, while those further away receive a slower airflow. To ascertain whatever effects different airflow speeds may have on the health of animals kept in stalls located at increasing distances from ventilation fans, we divided a select group of 43 Holstein dairy calves into six subgroups based on age, and each subgroup was subjected to either a specified high-speed or low-speed airflow as follows: (1) Six 3-day-olds received high-speed airflow (D3-HA); (2) Six 3-day-olds received low-speed airflow (D3-LA); (3) Eight 19 (±3)-day-olds received high-speed airflow (D19-HA); (4) Eight 19 (± 3)-day-olds received low-speed airflow (D19-LA); (5) Eight 29 (±3)-day-olds received high-speed airflow (D29-HA); and (6) Seven 29 (±3)-day-olds received medium-speed airflow (D29-MA). These trials show that the rectal temperatures and respiratory rates of D19-LA (39.37 °C; 72.90 breaths/min) were significantly higher than those of D19-HA (39.14 °C; 61.57 breaths/min) (*p* ≤ 0.05), and those of D29-MA (39.40 °C; 75.52 breaths/min) were significantly higher than those of D29-HA (39.20 °C; 68.41 breaths/min) (*p* ≤ 0.05). At 33 (±3) days of age, those calves receiving high-speed airflow (*p* ≤ 0.05) registered significantly higher immunoglobulins A and M than calves receiving low-speed flow. Those calves subjected to a high-speed airflow also registered significantly lower tumor necrosis factor levels than those receiving low-speed flow (*p* ≤ 0.05). Among the 29 to 43-day-old calves, no significant differences in immunity parameters were found to exist between groups D29-HA and D29-MA. On the basis of these findings, we were able to conclude that in the warm season, when the calves were less than 0.5 months old, low-speed (0.17–0.18 m/s) airflows had no significant effect on calves; when the calves were 1 month old, low-speed airflow (0.20–0.21 m/s) may impair the immune functions; when the calves were 1 to 1.5 months old, the airflow velocity higher than 0.9 m/s can meet the needs of the calf without a negative impact on the calf.

## 1. Introduction

The barns used to house dairy calves can usually be successfully ventilated either by a passive system that relies on naturally occurring airflows or an active system that uses electrically powered fans to regulate the flow of air through the barn. Active systems become especially relevant during periods of warm weather when the ambient temperature exceeds the animal’s upper critical temperature [1]. When insufficient cooling is achieved by opening doors, windows, and roof-ridge vents, applying other measures, such as a supplementary active ventilation system, should be employed to prevent the resulting “heat stress”. One such system that employs axial-flow fans is often used in the naturally ventilated barns that house adult dairy cows [2] and the barns housing dairy calves [3]. Axial-flow fans increase the speed of airflow and therefore can more quickly remove convective heat from the surface of a calf [4]. Moreover, as the velocity of the air passing over the calf increases, the insulation value of the animal’s coat will decrease and, thus, the animal will experience increased heat loss [5]. In turn, the animal will most likely suffer less of the heat stress that overheating can cause. The thermoneutral zone for a 1-month-old calf was estimated at 10–25 °C with an upper limit of 30 °C maximum acclimatable temperature, and a calf would be less susceptible to heat stress than an adult cow [6,7].

Calves housed individually are less likely to be exposed to infectious agents than those raised in group pens [8]; hence, the practice of raising calves individually now predominates in the dairy industry in some countries. For instance, in southern Brazil, 56% of the calves raised indoors are housed individually [9], while in Quebec, Canada, 89.7% are raised in this way [10]. The solid partitions between and in front of the calf pens prevent nose-to-nose contact and control drafts in an enclosed building [11]. Most of these facilities are ventilated by mechanical systems, and fan-driven systems can work well in group-housing situations; however, in some milking parlors equipped with mechanical ventilation, the cows themselves are baffles during the milking process [12]. A similar phenomenon occurs if the fans are arranged to propel air in a direction perpendicular to the compartments divided by solid partitions; the airflow will only benefit those calves in the first few pens, because the partitions act as barriers, deflecting the airflow away from stalls and further downstream [13]. Consequently, clean air often does not circulate fast enough around calves further from the fans. However, it should be noted that a separate study found that the presence of such panels correlates significantly with lower levels of respiratory disease in winter [14]. Removing the panels increases the speed of the airflow through the barn but also eliminates the protective effect the panels provide. To resolve the controversy raised by these findings, we investigated the effects that solid and mesh panels arrayed perpendicular to the direction of airflow may have on the wellbeing of the calves in our study. By quantifying these effects based on physiological performance and immunity parameters, we were able to propose a more suitable method for ventilating the barns in which dairy calves are typically raised.

Rectal temperature and respiratory rate are essential physiological performance indicators of thermal load [4,15] and health in cattle. Many factors can adversely affect a calf’s passive immunity and lead to a suppressed immune system [7], a condition that can be detected by measuring the animal’s serum immunoglobulin levels (IgG, IgA, and IgM) [16]. Furthermore, as the animal’s tissues become inflamed, its tumor necrosis factor (TNF-α) levels rise. Therefore, the increase in cytokine TNF-α, which plays an important role in an animal’s ability to regulate its immune and endocrine systems, is a marker of inflammation in cattle [17].

To our knowledge, no research focusing on calf-rearing systems has sought to determine the effects of axial fans and solid partitions on calves. All that is known is that calves in different stalls receive air at different speeds. This dearth of information and understanding also served as an impetus for this study.

## 2. Materials and Methods

### 2.1. Experimental House and Calf Management

**Figure 1:**
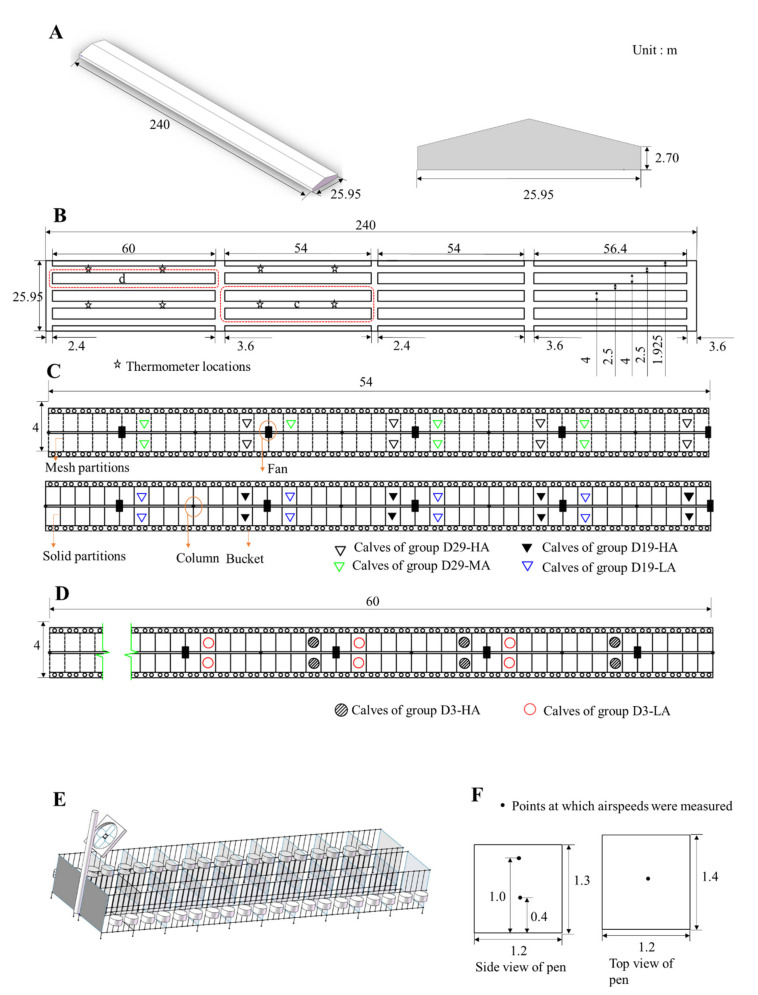
A schematic of the experimental calf house. (**A**) Calf house; (**B**) plan of the experimental house; (**C**) detailed plan of pens for 29 (±3)-day-old and 19 (±3)-day-old calves; (**D**) detailed plan of pens for 3-day-old calves; (**E**) detailed profile of calf pens; and (**F**) airflow speed measurement points in tested pens.

The present study was conducted in a calf house (32°34′ N, 120°54′ E) (Figure 1) located at a commercial Holstein dairy farm in the Jiangsu Province of China over a 2-week period (5 September to 19 September 2019). The calf house had a natural ventilation system augmented with axial fans (Shanghai Terrui Mechanical Equipment Co., Ltd., Shanghai, China). The natural vents were located on both sides of the sidewall; they were 2 m high and ran the length of the cowshed. The fans (diameter = 0.97 m) are usually operated at full speed (25,430 m^3^ h^−1^, 410 W, 720 rpm).

Each fan was installed on a column (located at 12 m intervals) at a height of 2.2 m and angled downward from the vertical at about 38° (Figure 1). The fan system was manually controlled and switched on when the ambient temperature inside the barn reached 20 °C. All the calves were fed colostrum three times on the day they were born, after which they were separated from their dams and placed inside individual pens (1.2 × 1.4 × 1.3 m) in an open calf house (240 × 25.95 × 2.7 m), and each pen was supplied with 5 cm of sawdust to serve as bedding. Detachable planks formed the walls that separated the pens, and to sustain each calf, two stainless steel buckets were used—one to supply concentrated feed and the other to supply milk and water. All the calves were provided milk manually at 07:00, 14:00, and 19:00 every day. The milk fed to the calves was provided according to this schedule: 6 L per day from days 1 to 4; 9 L per day from days 15 to 24; 12 L per day from days 25 to 44; 9 L per day from days 45 to 54; and 6 L per day from days 55 to 61.

### 2.2. Experimental Design

**Figure 2:**
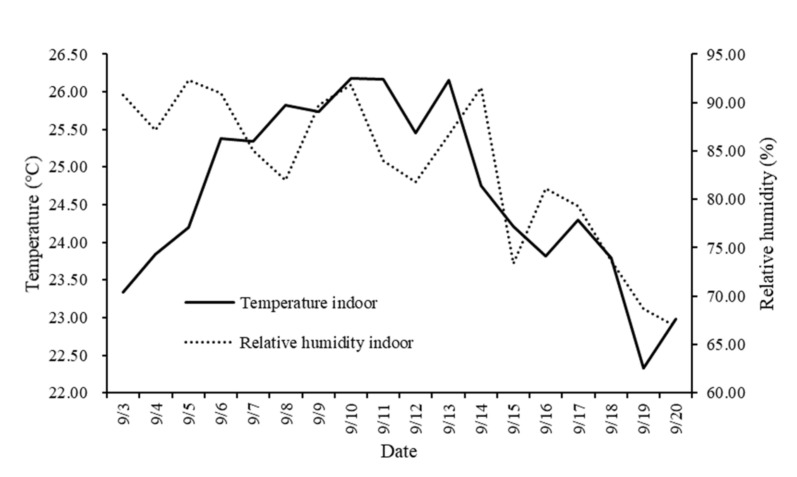
Average daily temperature and relative humidity inside the experimental house.

**Figure 3:**
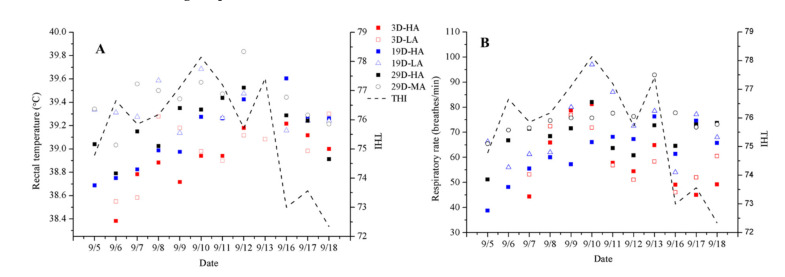
Daily changes (mean ± SEM) of rectal temperature (**A**) and respiratory rate (**B**) with average daily THI of groups 3D-HA, 3D-LA, 19D-HA (19 (±3)-day-olds who received high-speed airflow), 19D-LA (19 (±3)-day-olds who received low-speed airflow), 29D-HA (29 (±3)-day-olds who received high-speed airflow), and 29D-MA (29 (±3)-day-olds who received medium-speed airflow). THI is the temperature and humidity index.

**Figure 4:**
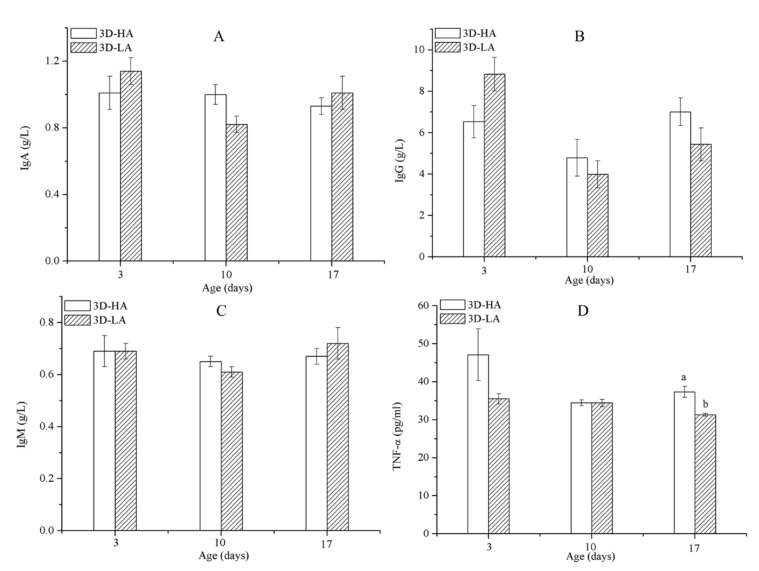
Immunoglobulin levels IgA (**A**), IgG (**B**), IgM (**C**), and tumor necrosis factor (TNF)-α (**D**) concentrations among calves of D3-HA and D3-LA. Data represent mean ± SEM. ^a,b^ Values having different letters within the same column are significantly different (*p* ≤ 0.05).

**Figure 5:**
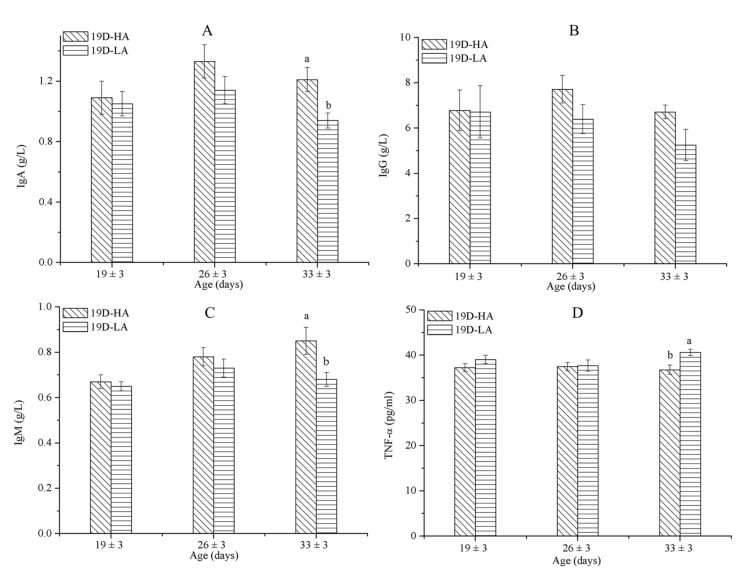
IgA (**A**), IgG (**B**), IgM (**C**), and TNF-α (**D**) concentrations among calves of D19-HA and D19-LA. Data represent mean ± SEM. ^a,b^ Values having different letters within the same column are significantly different (*p* ≤ 0.05).

**Figure 6:**
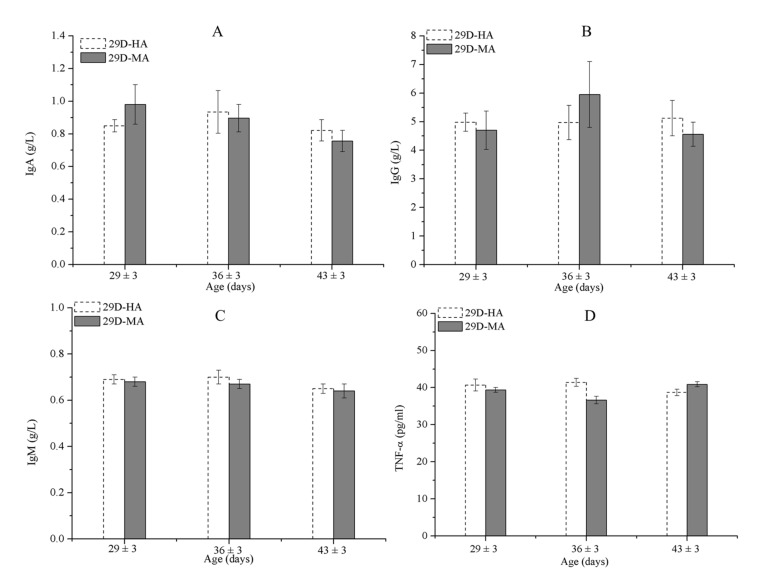
IgA (**A**), IgG (**B**), IgM (**C**), and TNF-α (**D**) concentrations among calves in D29-HA and D29-MA. Data represent mean ± SEM.

Forty-three female calves were selected and divided into six groups, with each group treated with a specified ventilation rate: (1) Group D3-HA calves were 3 days old and subjected to a high-speed airflow (N = 6 calves) (Figure 1D); (2) Group D3-LA were 3 days old and subjected to a low-speed airflow (N = 6 calves) (Figure 1D); (3) Group D19-HA were 19(±3) days old and subjected to a high-speed airflow (N = 8 calves) (Figure 1C); (4) Group D19-LA were 19(±3) days old and subjected to a low-speed airflow (N = 8 calves) (Figure 1C); (5) Group D29-HA were 29 (±3) days old and subjected to a high-speed airflow (N = 8 calves) (Figure 1C); (6) Group D29-MA were 29 (±3) days old of age and subjected to a medium-speed airflow (N = 7 calves) (Figure 1C). Each calf was fed 4 L of colostrum within 2 h after birth and 2 L after 6-8 h after birth. Each were separated from its dam after birth. The calves’ total serum protein concentrations were tested on day 3 and were found to be over 5.2 g/L (Table 1). A total serum protein concentration > 5.2 g/L is often considered indicative of an adequate passive transfer of immunity in healthy, well-hydrated calves [18]. For groups D3-HA, D3-LA, D19-HA, and D19-LA, the calves in the solid-partitioned stalls closest to the fans received the highest speed airflow, while those in pens furthest from the fans received the lowest speed airflow. For groups D29-HA and D29-MA, the solid partitions were replaced with mesh partitions before the beginning of the test (2 September 2019), allowing air to circulate freely between stalls.

Air temperature and relative humidity within the calf house were automatically measured at 1-min intervals by eight digital data loggers (HOBO U23 Pro v2; Onset Computer Co. Ltd., Bourne, MA, USA; temperature accuracy of ±0.21 °C, range 0 to 50 °C; relative humidity accuracy of ±5% from 10% to 90%) located 1.5 m above the floor (Figure 1B). The temperature-humidity index (THI) was calculated using the formula [19]: THI = (1.8 *T_a_* + 32) − 0.55 (1 − 0.01 *RH*) × (1.8 *T_a_* − 26), *T_a_* being ambient temperature (°C) and *RH* being relative humidity (%). Airspeed was measured by averaging the readings for 3 min using a multifunctional heat-wire anemometer (9565-P with 964 probe, TSI Inc., Shoreview, MN, USA; ±3% of reading, 0.01 m/s resolution). The airflow inside the pens was monitored at heights of 0.4 m and 1.0 m above the floor (1.0 m is the average height of a standing calf’s back and 0.4 m is the height of an average calf’s back when lying down; both heights are typically monitored when measuring the airflow inside in a calf pen) (Figure 1F). At each height, the airflow speed was measured in the middle of the pen. The airflow speeds were monitored for 4 days for groups D3-HA and D3-LA on September 7, 9, 11, 17, and the airflows for groups D19-HA, D19-LA, D29-HA, and D29-MA were monitored for 4 days on 5, 7, 9, and 11 September. 

### 2.3. Respiratory Rates, Rectal Temperatures, ADGs, and Blood Collection and Measurement

Respiratory rates were measured with the aid of a stopwatch twice daily at 10:00–10:30 and 15:00–15:30. The respiratory rate was tallied by counting the number of times the animal’s abdominal muscles expanded and relaxed during the measurement period while the calf was in the prone position. The rectal temperature of each calf was measured at 15:00 each day using a digital thermometer, which was checked and calibrated at the start of the test. The weight of the calf was measured at birth and at the end of the test. The average daily gain (ADG) achieved by each calf was calculated by subtracting the animal’s birth weight from its weight at the end of the test and dividing the difference by the animal’s age (in days).

Blood samples were collected three times (5, 12, 19 September 2019) via jugular venipuncture. Samples were analyzed for IgA, IgG, IgM, and TNF-α concentrations. To determine total concentrations, blood samples were collected using a vacuum blood collector (without anticoagulant), and the samples were kept at room temperature (28 °C) until the blood clotted; then, the serum was separated by centrifugation at 4000× *g* for 3 min, aliquoted, and stored frozen (−20 °C) until they could be assayed. The concentrations of IgA, IgG, IgM, and TNF-α were detected using commercially available Enzyme-Linked Immunosorbent Assay (ELISA) kits (Beijing Kang Jia Hong Yuan Biological Technology Co., Ltd., Beijing, China).

### 2.4. Illness Diagnose and Define

Illness were defined and diagnosed by a qualified veterinarian. Table 2 lists the health criteria and explains the scoring system. When a calf registered a fecal score of 2 or 3, diarrhea was the diagnosis. Respiratory disease was the diagnosis when a calf registered cough score of 2 or 3, and either nasal discharge (score of 1, 2 or 3) or ocular discharge (score of 1, 2, or 3) [20].

### 2.5. Statistical Analysis

All analyses were performed using SPSS software version 17.0 (IBM Corp., Armonk, NY, USA). Differences between treatments were deemed statistically significant if the associated *p*-value ≤ 0.05. The immunity parameters data were analyzed using linear mixed models. The data were analyzed according to the fixed effects of airspeed and the ages of calves. The model’s governing equation was as follows:

*Yij_k_* = *μ* + *AS_i_* + *DA_j_* + *Rk* + *AS_i_* × *Age_j_* + *εij_k_*

where *Yij_k_* = parameters investigated; *μ* = model constant; *AS_i_* = effect of airspeed (*i* = 1 to 2); *Age_j_* = effect of age (days) (*j* = 1 to 3); *Rk* = replicate; *WS_i_* × *Age_j_* = effect produced by the interaction of airspeed and age; *εij_k_* = the residual error term.

## 3. Results

### 3.1. Temperature Inside the Experimental House

Throughout the experiment, the average daily ambient temperature inside the experimental house ranged from 22.33 to 26.18 °C, and relative humidity ranged from 66.90% to 92.28% (Figure 2).

### 3.2. Airspeeds in the Calf Pens

The data for airspeed are shown in Table 3, Table 4, Table 5 and Table 6. Table 3 shows that the airspeeds at the same height did not differ significantly during the 4 days. In other words, during the experiment, each group was essentially subjected to a steady airflow. Significant differences in airflow were detected at the same heights across the six groups (Table 4, Table 5 and Table 6). The airflow speeds recorded at 0.4 m and 1.0 m for the groups subjected to high airspeeds were significantly greater than the other groups (D3-HA vs. D3-LA; D19-HA vs. D19-LA; D29-HA vs. D29-MA) (*p* ≤ 0.05).

### 3.3. Rectal Temperature, Respiratory Rate, and ADG of Tested Calves

A calf’s rectal temperature and respiratory rate will vary depending, to some extent, on the current level of THI as well as the speed at which air passes over the animal (Table 3, Table 4, Table 5 and Figure 3). It should be noted that the welfare of a young calf becomes compromised when the THI rises above 78, and when the THI has risen to 88 or above, the calf will begin to experience significant heat-related stress [21]. In this study, the THI remained below 78 in the calf house; thus, the calves were never in a state of heat stress during this period. Rectal temperature and respiratory rate tend to increase as the THI rises, and they tend to decrease as the THI declines. The rectal temperatures and respiratory rates observed for D19-LA were significantly higher than those for D19-HA (*p* ≤ 0.05) (Table 4), and those for D29-MA were significantly higher than those for D29-HA (*p* ≤ 0.05) (Table 5). However, the rectal temperatures and respiratory rates of D3-HA and D3-LA did not differ significantly (Table 3). In addition, the ADG did not differ significantly among those groups.

### 3.4. Immunity Parameters and Health Status of Calves Subjected to Different Airspeeds

As shown in Table 7, the combination of airspeed and age significantly affected the concentrations of IgG and TNF-α (*p* ≤ 0.05) in D3-HA and D3-LA. Furthermore, the TNF-α levels of the calves decreased as they aged from 3 to 17 days old; when measured at 17 days of age, the TNF-α concentrations for calves provided a high airspeed were significantly greater than those for calves provided a low airspeed (*p* ≤ 0.05) (Figure 4D). Airspeed had no significant effect on the serum concentrations of IgA, IgM, or IgG in calves of the same age (Figure 5). During the experiment, the morbidities were 5% in group D3-HA and 2% in group D3-LA (Table 8). These results indicate that the immune systems of calves under 17 days of age were negatively affected by being subjected to a high airflow speed from the day they were born.

As shown in Table 9, the combination of airspeed and age significantly affected the concentrations of IgA and IgM among the calves in D19-HA and D19-LA (*p* ≤ 0.05). Moreover, it appears that airspeed significantly affected the concentrations of IgA, IgM, and TNF-α (*p* ≤ 0.05). At 33 (±3) days old, the concentrations of IgA and IgM among the calves subjected to a high airflow speed (*p* ≤ 0.05) were significantly greater than the concentrations of those exposed to a low airflow speed (Figure 5A,C); the TNF-α associated with a high airflow speed was significantly less than that associated with a low airflow speed (*p* ≤ 0.05) (Figure 5D). In contrast, airflow speed will not affect the serum concentrations of IgG in calves of the same age. During the experiment, the morbidities in groups D19-LA and D19-HA were 10% and 0%, respectively (Table 8). The immunity parameter results agreed with the prevalence of illness in the calves.

The solid partitions separating 27-day-old calves were replaced by mesh partitions, and the results show there were no significant differences in immunity parameters between D29-HA and D29-MA (Figure 6 and Table 10), and the morbidity in both groups was 0% (Table 8). Therefore, replacing solid partitions with mesh partitions did not increase the risk of illness in 27-day-old calves.

## 4. Discussion

### 4.1. Rectal Temperature, Respiratory Rate, and ADG of Young Animals Subjected to Different Airspeeds

Many studies have shown that the core body temperature is significantly influenced not only by the ambient temperature but also the airspeed, relative humidity, and solar radiation levels that predominate in an animal’s surrounding environment [22,23]. Moreover, these factors all work in concert. Accordingly, we found that a young diary calf’s rectal temperature and respiratory rate are significantly affected by both the ambient temperature and the velocity of the airflow passing over the animal: those calves we subjected to a relatively high airspeed all registered lower rectal temperatures and respiratory rates than the calves subjected to a low airspeed (however, airspeed does not appear to have any significant effect on a calf’s average daily weight gain). Additionally, when a calf becomes ill, its core body temperature and breathing rate can show as abnormal.

During the first 2 months of an average Holstein calf’s life, its rectal temperature ranges from 38.1 to 39.2 °C [24], and the thermoneutral zone of calves less than 21 days of age ranges from 15 to 25 °C [25]. Since they had not been subjected to any heat stress, the rectal temperatures and respiratory rates of the calves in group D3-HA were found to be similar to those in D3-LA (*p* ≤ 0.05). When the calves were 19–44 days old, the high airspeed decreased the respiratory rate and rectal temperature compared with the low/medium speed. It seems reasonable to conclude that increasing the flow airspeed increase the heat flux and amount of heat released from a cow; thus, a higher flow speed produces a larger convective-heat-transfer coefficient on the surface of the cow [26]. If this is true, then a faster flow could affect the animal’s welfare. In a recent study involving bulls kept inside a roofed structure, ceiling fans (which produced an airflow velocity of 6.61 m/s) resulted in a higher percentage of dry straw bedding and consequently cleaner bulls, when compared to results produced by axial fans (which produced an airflow velocity of 1.71 m/s) [27]. However, it should be noted that if the airspeed is too great, the animals may be unduly irritated and be forced to shed excess body heat. It can also raise dust and scattering feed [26]. A similar study that looked at the physiological changes in calves housed under different flow rates found that the skin surface temperatures of the tested animals declined when airspeeds increase, and it also found that changing the flow velocity from “still” to 1 m/s reduced the temperature of dry skin by 0.5 °C [28]. Once the calf hutches were elevated, the airspeed consequently increased (from 2.1 to 6.2 km/h), and calves registered lower respiratory rates [29]. Therefore, based on these findings, it appears that maintaining an adequate airspeed in an area designed to house dairy calves should be a key component of any strategy aimed at reducing the negative physiological responses associated with heat and hygiene. Moreover, providing adequate airspeed could help to increase a young calf’s threshold temperature (the temperature at which the animal’s respiratory rate exceeds 53% of maximum) and reduce the negative effects of relative humidity [30].

A rise in rectal temperature of 1 °C or less is enough to reduce performance in most species [31,32]. Notably, we found no differences in the ADGs of calves of the same age subjected to different airflow speeds in our study. That the ADGs recorded during this study were higher than those reported in the literature suggests that the calves used in this study were raised well (a meta-analysis of pre-weaned calves showed that the ADG was 0.52 ± 0.13 kg) [33]. In addition, the ADG achieved by a calf from birth to 3 weeks of age (from week 0 to 3) and from 3 to 6 weeks of age normally ranges from 0.18 to 0.26 kg and 0.56 to 0.69 kg, respectively [34]).

### 4.2. Immunity Parameters and Health Status of Young Animals of Various Ages Subjected to Different Airflow Speeds

A high level of immunoglobulins, which may be caused by a highly contaminated environment and commensurately high risk of infection, can actually promote the development of a more active immune system. On the other hand, a high-speed airflow usually provides air that is cleaner and therefore healthier than still air. Thus, it can be concluded that higher levels of immunoglobulins will produce a stronger immune system.

An animal’s immunoglobulin levels can decrease because it receives insufficient amounts of milk from its mother or because of an impaired ability to produce endogenous immunoglobulins, and the levels can be elevated by an ongoing infection. Thus, when determining the effects of airspeed, the levels of IgA, IgM, and TNF-α should be measured. Accordingly, when we analyzed the data for calves subjected to a slow airflow from day 19–33, we found they had lower levels of serum IgA and IgM and higher levels of TNF-α than the calves subjected to a fast airflow. For calves of D29-HA and D29-MA, the solid partitions were replaced by mesh partitions; accordingly, the pens farthest from the fans received a medium airflow speed. There were no significant differences in the IgA, IgM, and TNF-α levels between groups D29-HA and D29-MA. Meanwhile, a previous study showed that serum IgA concentrations peaked on day 1 and then declined until day 14, at which time they began to stabilize (0.7 ± 0.06 g/L) [35]. The study showed that the calves showed approximately the same amounts of serum IgA and IgM regardless of whether they were subjected to fast or slow airflow. However, the immune function (as indicated by both IgA and IgM levels) of calves subjected to a slow airflow for 33 (±3) days was significantly lower than that of calves subjected to a fast airflow (*p* ≤ 0.05). Moreover, the TNF-α levels of calves subjected to a slow airflow decreased from days 3 to 17 and increased from days 19 to 33. Perhaps individual differences among the animals contributed to these effects, as the calves tested at 3 days of age and those tested at 19 days were different animals. Furthermore, a low-velocity airflow administered during warm weather will affect a calf’s immune system if the calf is more than 1 month old. Of the calves that were tested at 33 (±3) days of age, those subjected to a fast airflow registered significantly lower TNF-α levels than the calves subjected to a slow airflow (*p* ≤ 0.05). We should also note that a faster airflow can more rapidly disperse the harmful gases produced by feces and urine and encourages the evaporation of urine from the animal’s bedding.

The calves’ IgG levels did not vary significantly from day 3 and throughout the test period, regardless of the airspeed. Several studies have demonstrated that calves with serum IgG concentrations of >10 g/L or total serum protein >5.2 g/L show lower morbidity and mortality rates during the preweaning period [36,37,38]. Conversely, calves with serum IgG concentrations of ≤10 g/L are at a greater risk of contracting disease; therefore, a target serum IgG concentration of 15 g/L has been suggested as the optimal maintenance level to significantly reduce morbidity and mortality during the preweaning period [39]. Meanwhile, our results show that the concentration of serum IgG did not change significantly among calves subjected to fast and slow airflows (*p* > 0.05).

It is generally accepted that contact between individual calves increases the risk of disease. However, reports have shown that tactile contact, pair housing, or small-group housing improves calf welfare and does not increase the risk of diarrhea and respiratory disease [40,41,42]. The mesh partitions used in the pens housing groups D29-HA and D29-MA allowed tactile and visual contact, and those calves were healthy during the experiment. Moreover, mesh partitions can improve the flow of air around the pens furthest from the fans. A study confirmed that housing calves in a group pen during the first month of life is disadvantageous compared with housing calves in individual pens [8].

### 4.3. Preferred Ventilation Systems

A newly developed ventilation system involves a positive-pressure polytube running the length of the barn’s center aisle that, at regular intervals, emits jets of air aimed directly down onto animals from above. The system has been tested by several researchers and found to work effectively. For example, Nordlund [43] found that a polytube system designed to operate during winter months reduced bacterial counts by approximately 25% and the incidence of respiratory disease by approximately 75%. Based on these as well as other findings, Nordlund and Halbach [13] were able to conclude that a natural ventilation system supplemented with a positive-pressure polytube system provides the optimal cooling effect. In the same study, Nordlund and Halbach provided suggestions for the ventilation rate, tube diameter, tube height above the floor, and the optimal spacing between jet emitters. Mondaca and Choi [44] subsequently concluded that a positive pressure ventilation system (appropriately designed, installed, and managed) can effectively cool individual animals. A previous study also investigated the effects of three key parameters (air-supply speed, distribution tube diameter, and air-supply angle) on the performance of a positive-pressure polytube ventilation system on a standing and prone cow [45]. The study showed that a positive pressure tube system ensured that all dairy cows in every stall of a naturally ventilated barn had a sufficient amount of fresh air. In light of the findings of these earlier studies, researchers should seek to determine the effects of applying a positive-pressure polytube ventilation system in houses used to shelter other commercially raised animals.

## 5. Conclusions

In this study, we sought to compare the physiological and immune health of dairy calves housed in separate stalls under one roof, and these calves were subjected to an axial-flow fan ventilation system at one end of the barn propelling air perpendicular to the stalls. Our investigation involved two groups of female Holstein calves; one group was kept in stalls located farthest from the axial-flow fans, and the other was kept close to the fans.

While the applications of the study may have some limitations due to the lack of individual pen temperature and relative humidity data and no control group in the experiment, there were three major findings of this study: (1) When temperatures were between 22 and 26 °C in barn, the calves (aged 19 to 44 days) exposed to a high-speed airflow (1.5 to 3.0 m/s) demonstrated lower rectal temperatures and respiratory rates than those exposed to a low/medium-speed airflow (0.2/0.9 m/s). (2) The serum concentrations of IgA, IgG, and IgM of calves less than 17 days old were unaffected by both the high and the low airspeed; however, after the calves reached 17 days, the TNF-α concentrations of calves receiving a high-speed airflow were significantly greater than those of calves receiving a low-speed airflow. Calves subjected to a low-speed airflow for over 1 month showed lower levels of IgA and IgM and higher levels of TNF-α than the calves subjected to a high-speed airflow. (3) There were no significant differences in the serum IgA, IgG, and IgM concentrations among 29 to 44-day-old calves (housed in stalls separated with mesh partitions) under the high-speed or the medium-speed airflows.

Therefore, we concluded that in the warm season, when the calves were less than 0.5 months old, low-speed (0.17–0.18 m/s) airflows had no significant negative effect on calves; when the calves were 1 month old, low-speed airflow (0.20–0.21m/s) significantly reduce their immunity performance; and when the calves were 1 to 1.5 months old, the airflow velocity higher than 0.9 m/s was required to meet the needs of the calf without a negative impact on their health.

## Figures and Tables

**Table 1:** Total serum protein of tested calves.

Number of Calves	Total Serum Protein (g/L)					
	D3-HA	D3-LA	D19-HA	D19-LA	D29-HA	D29-MA
1	9.7	11.7	10.8	8.3	8.5	8.3
2	10.1	9.4	9.5	9.5	10.3	8.2
3	7.8	8.3	9.2	7.0	8.9	9.5
4	9.2	9.0	8.7	8.1	8.7	8.9
5	8.4	9.2	7.3	11.2	8.9	9.4
6	9.0	8.4	8.4	9.2	9.3	8.8
7	8.0	9.5	-	-	11.1	7.7
8	9.4	10.3	-	-	9.9	-

**Table 2:** Calf health scoring system.

Health Factor	Scoring System			
	0	1	2	3
Fecal score	Normal	Semi-formed, pasty	Loose, but stays on top of bedding	Watery, sifts through bedding
Eye score	Normal	Small amount of ocular discharge	Moderate amount of bilateral discharge	Heavy ocular discharge
Cough	Normal	Induce single cough	Induced repeated coughs or occasional spontaneous cough	Repeated spontaneous coughs

**Table 3:** Airspeeds (mean ± SEM) in calf pens.

Group	Height	Airspeed (m/s)				*p*-Value
		Day 1	Day 2	Day 3	Day 4	
D3-HA	1.0 m	2.52 ± 0.30	2.37 ± 0.35	2.59 ± 0.27	1.70 ± 0.21	0.20
	0.4 m	1.45 ± 0.45	1.22 ± 0.22	1.69 ± 0.44	1.06 ± 0.24	0.63
D3-LA	1.0 m	0.17 ± 0.02	0.18 ± 0.02	0.18 ± 0.02	0.19 ± 0.02	0.76
	0.4 m	0.17 ± 0.03	0.18 ± 0.02	0.16 ± 0.03	0.19 ± 0.01	0.78
D19-HA	1.0 m	2.62 ± 0.12	2.84 ± 0.13	2.97 ± 0.17	3.21 ± 0.16	0.07
	0.4 m	1.35 ± 0.20	1.63 ± 0.21	2.13 ± 0.31	1.96 ± 0.26	0.14
D19-LA	1.0 m	0.16 ± 0.01	0.21 ± 0.05	0.20 ± 0.05	0.21 ± 0.02	0.80
	0.4 m	0.18 ± 0.01	0.23 ± 0.06	0.17 ± 0.03	0.26 ± 0.04	0.25
D29-HA	1.0 m	2.04 ± 0.31	2.34 ± 0.32	2.65 ± 0.22	2.31 ± 0.30	0.53
	0.4 m	2.14 ± 0.25	2.19 ± 0.28	2.35 ± 0.12	2.35 ± 0.26	0.88
D29-MA	1.0 m	0.79 ± 0.11	1.12 ± 0.15	0.80 ± 0.08	0.92 ± 0.14	0.22
	0.4 m	0.86 ± 0.12	1.05 ± 0.15	0.76 ± 0.14	0.98 ± 0.10	0.40

**Table 4:** Airspeeds, respiratory rates, rectal temperatures, and average daily gain (ADG) (mean ± SEM) of calves of groups D3-HA and D3-LA.

Group	Airspeed (m/s)		Respiratory Rate (breaths/min) ^1^	Rectal Temperature (°C) ^1^	ADG (kg/d)
	0.4 m	1.0 m			
D3-HA	1.36 ± 0.17 ^a^	2.29 ± 0.16 ^a^	57.65 ± 2.34	38.90 ± 0.03	0.64 ± 0.09
D3-LA	0.17 ± 0.01 ^b^	0.18 ± 0.01 ^b^	59.98 ± 1.98	39.03 ± 0.05	0.67 ± 0.05

Note: Group D3-HA calves were 3 days old and subjected to a high airspeed; Group D3-LA were 3 days old and subjected to a low airspeed; ^a,b^ Values having different letters within the same column are significantly different (*p* ≤ 0.05). ^1^ The data of respiratory rates and rectal temperatures used only include data for healthy calves.

**Table 5:** Airspeeds, respiratory rates, rectal temperatures, and ADG (mean ± SEM) of calves of groups D19-HA and D19-LA.

Group	Airspeed (m/s)		Respiratory Rate (breaths/min) ^1^	Rectal Temperature (°C) ^1^	ADG (kg/d)
	0.4 m	1.0 m			
D19-HA	1.77 ± 0.13 ^a^	2.91 ± 0.08 ^a^	61.57 ± 1.80 ^b^	39.14 ± 0.05 ^b^	0.88 ± 0.04
D19-LA	0.21 ± 0.02 ^b^	0.20 ± 0.02 ^b^	72.90 ± 2.34 ^a^	39.37 ± 0.05 ^a^	0.84 ± 0.02

Note: Group D19-HA were 19 (±3) days old and subjected to a high airspeed; Group D19-LA were 19(±3) days old and subjected to a low airspeed; ^a,b^ Values having different letters within the same column are significantly different (*p* ≤ 0.05). ^1^ The data of respiratory rates and rectal temperatures used only include data for healthy calves.

**Table 6:** Airspeeds, respiratory rates, rectal temperatures, and ADG (mean ± SEM) of calves of groups D29-HA and D29-MA.

Group	Airspeed (m/s)		Respiratory Rate (breaths/min) ^1^	Rectal Temperature (°C) ^1^	ADG (kg/d)
	0.4 m	1.0 m			
D29-HA	2.26 ± 0.11 ^a^	2.35 ± 0.14 ^a^	68.41 ± 1.70 ^b^	39.20 ± 0.05 ^b^	0.80 ± 0.04
D29-MA	0.91 ± 0.06 ^b^	0.91 ± 0.04 ^b^	75.52 ± 1.65 ^a^	39.40 ± 0.05 ^a^	0.81 ± 0.04

Note: Group D29-HA were 29 (±3) days old and subjected to a high airspeed; Group D29-MA were 29 (±3) days old of age and subjected to a medium airspeed. ^a,b^ Values having different letters within the same column are significantly different (*p* ≤ 0.05). ^1^ The data of respiratory rates and rectal temperatures used only include data for healthy calves.

**Table 7:** Immunity parameters of calves in D3-HA and D3-LA.

Item ^1^	IgA (g/L)	IgG (g/L)	IgM (g/L)	TNF-α (pg/mL)
AS				
High	0.98	6.11	0.67	39.62 ^a^
Low	0.91	6.08	0.67	33.73 ^b^
SEM	0.05	0.48	0.02	1.77
Age (days)				
3	1.08	7.68 ^a^	0.69	41.29 ^a^
10	0.91	4.39 ^b^	0.63	34.43 ^b^
17	0.97	6.22 ^a^	0.70	34.30 ^b^
SEM	0.06	0.59	0.03	2.17
*p*-value				
AS	NS ^2^	NS	NS	≤0.05
Age	NS	≤0.05	NS	≤0.05
AS × Age	NS	≤0.05	NS	≤0.05

Note: ^a,b^ Values having different letters within the same column are significantly different (*p* ≤ 0.05); ^1^ AS = airspeed; ^2^ NS = no significance.

**Table 8:** Morbidity of calves during the experiment.

Item	D3-HA	D3-LA	D19-HA	D19-LA	D29-HA	D29-MA
Days × number	120	120	160	160	160	140
No. days of illness	6	2	0	16	0	0
Percentage of illness days	5%	2%	0%	10%	0%	0%

**Table 9:** Immunity parameters of calves in D19-HA and D19-LA.

Item ^1^	IgA (g/L)	IgG (g/L)	IgM (g/L)	TNF-α (pg/mL)
AS				
High	1.21 ^a^	7.07	0.77 ^a^	37.16 ^b^
Low	1.04 ^b^	6.12	0.69 ^b^	39.11 ^a^
SEM	0.05	0.43	0.02	0.56
Age (days)				
19 ± 3	1.07	6.75	0.66 ^b^	38.15
26 ± 3	1.24	7.05	0.76 ^a^	37.58
33 ± 3	1.07	5.98	0.77 ^a^	38.67
SEM	0.06	0.53	0.03	0.69
*p*-value				
AS	≤0.05	NS ^2^	≤0.05	≤0.05
Age	NS	NS	≤0.05	NS
AS × Age	≤0.05	NS	≤0.05	NS

Note: ^a,b^ Values having different letters within the same column are significantly different (*p* ≤ 0.05); ^1^ AS = airspeed; ^2^ NS = no significance.

**Table 10:** Immunity parameters of calves in D29-HA and D29-MA.

Item ^1^	IgA (g/L)	IgG (g/L)	IgM (g/L)	TNF-α (pg/mL)
AS				
High	0.93	5.02	0.68	40.27
Medium	0.94	5.07	0.66	38.97
SEM	0.04	0.26	0.01	0.44
Age (days)				
29 ± 3	0.98	4.84	0.68	40.03
36 ± 3	0.98	5.46	0.69	39.03
43 ± 3	0.84	4.83	0.65	39.79
SEM	0.07	0.44	0.02	0.76
*p*-Value				
AS	NS ^2^	NS	NS	NS
Age	NS	NS	NS	NS
AS × Age	NS	NS	NS	≤0.05

Note: ^1^ AS = airspeed; ^2^ NS = no significance.

## Data Availability

The data presented in this study are available on request from the corresponding author.
